# Application of Mass Spectrometry for Determining the Geographic Production Area of Wagyu Beef

**DOI:** 10.3390/metabo12090777

**Published:** 2022-08-23

**Authors:** Shuji Ueda, Yasuharu Takashima, Yunosuke Gotou, Ryo Sasaki, Rio Nakabayashi, Takeshi Suzuki, Shinji Sasazaki, Ituko Fukuda, Biniam Kebede, Yuki Kadowaki, Maiko Tamura, Hiroki Nakanishi, Yasuhito Shirai

**Affiliations:** 1 Department of Agrobioscience, Graduate School of Agricultural Science, Kobe University, Hyogo 657-8501, Japan; 2 Incorporated Administrative Agency Food and Agricultural Materials Inspection Center, Saitama 330-0081, Japan; 3 Food Oil and Fat Research Laboratory, Miyoshi Oil & Fat Co., Ltd., Tokyo 124-8510, Japan; 4 Department of Food Science, University of Otago, P.O. Box 56, Dunedin 9054, New Zealand; 5 Lipidome Lab Co., Ltd., Akita 010-0825, Japan

**Keywords:** Japanese Black cattle, metabolomics, origin determination, Hybrid Wagyu, triolein

## Abstract

Japanese Black cattle (Japanese Wagyu) beef is attracting attention for its aroma and marbling, and its handling is increasing worldwide. Here, we focused on the origin discrimination of Wagyu beef and analyzed the nutritional components of Japanese Wagyu (produced in multiple prefectures of Japan), Hybrid Wagyu (a cross between Angus and Wagyu cattle born in Australia and transported to Japan), and Australian Wagyu beef using mass spectrometry (MS). Triple-quadrupole liquid chromatography–MS was used to clarify the molecular species of lipids in Wagyu beef. Fourteen classes of lipids were separated, and 128 different triacylglycerides (TGs) were detected. A simple comparative analysis of these TGs using high-performance liquid chromatography revealed significantly higher levels of triolein (C18:1/C18:1/C18:1; abbreviated OOO) and C18:1/C18:1/C16:1 (OOPo) in Japanese Wagyu. Wagyu elements beef were comprehensively analyzed using inductively coupled plasma (ICP)–MS and ICP–optical emission spectrometry. We found significant differences in the rubidium, cesium, and lithium levels of Japanese and Australian Wagyu beef. On comparing metabolites using gas chromatography–MS, we identified significant differences in the levels of amino acids and other components of the Japanese and Australian Wagyu beef. These results suggest the possibility of determining the origin of Wagyu cattle breeds using MS and genetic discrimination.

## 1. Introduction

The global population is projected to reach 9.8 billion by 2050, and livestock production is expected to increase by 455 million tons until 2050. One solution to this challenge is establishing a system for sustainable food production. However, improving the productivity of agricultural products suitable for local environments is challenging. The efficient crossborder trade of livestock products can reduce global food-production challenges [1,2,3]. The primary livestock product exported by Japan is the Japanese Black cattle or the Japanese Wagyu. Japanese Wagyu is an original breed that is only found in Japan [4]. The meat from this cattle is characterized by excellent marbling derived from the intramuscular fat [5,6], tender meat, and a sweet aroma (Wagyu beef aroma) [7,8,9]. Japanese Wagyu evolved as a native breed and has adapted to Japan’s unique climate and environment. In medieval Japan, Wagyu cattle were widely used for rice farming and cargo transport. The current Japanese Wagyu is a descendent of the Tajiri strain in Hyogo Prefecture, and it has inherited the genes for good meat quality [4]. Overall, 2.55 million Japanese Wagyu cattle were produced in 2020 [10].

Consumer sensory testing has revealed that the fat content responsible for marbling is correlated with favorable beef flavor [11,12,13]. Interest in Wagyu beef has spread to the premium market [14,15,16]. Currently, Wagyu beef is also produced outside Japan [17]; Wagyu production in Australia, USA, China, UK, New Zealand, and other countries reached approximately 400,000 cattle in 2020 [18]. 

International law mandates the tracking and certification of food ingredients and the regions in which they are processed and manufactured by food-safety management. The European Union has implemented relevant laws that protect the names of products linked with the production region, including Protected Designation of Origin and Protected Geographical Indication [19,20,21]. The most common unfair competitive practice for agricultural products is the marketing of low-priced products as high-priced products [22]. Geographical indication (GI) systems are used globally to protect farmers from imitation, counterfeiting, and unfair competition for agricultural products. GI tags have been registered for Japanese Black cattle reared in specific regions of Japan, i.e., Hyogo, Shiga, Miyazaki, Kagoshima, Yamagata, Iwate, and Hiroshima Prefectures [4].

Food certification is a procedure for verifying product and labeling conformity and compliance with applicable legal and regulatory provisions. However, food fraud occurs in various forms, including brand imitation, reduced manufacturing costs, and extended shelf life. Food fraud represents an economic and potential food-safety issue [23,24]. Therefore, developing an instrumental analysis technique to authenticate products and a quick, inexpensive, and reliable method for food certification is crucial [25].

Recently, genetic analysis of food products has been used to identify the breed of livestock products; however, this method is inadequate to determine the origin of livestock products that have extremely similar DNA sequences [26]. Therefore, as an alternative, mass spectrometry (MS) has been used to prove the geographical origin of food products [27]. MS-based analyses in food evaluation include liquid chromatography–tandem MS (LC–MS/MS) [28], gas chromatography–MS (GC–MS), inductively coupled plasma (ICP)–MS, and ICP–emission spectrometry (OES) [29]. Depending on the characteristics of the food component, GC–MS (targeting organic acids, sugars, and amino acids), LC–MS/MS (targeting nonvolatile components), ICP–MS, and ICP–OES (targeting elemental components) are used. Furthermore, combining high-resolution MS with LC–MS/MS may provide more accurate information on complex components, such as lipids [30]. Such MS-based techniques improve the analytical accuracy and provide high-throughput screening compared with conventional chemical analytical methods.

Foods contain unique elemental components that vary with the producing region’s feed, water, and soil. Foods also have characteristic distributions of metabolites and lipids that depend on the breed. MS-based metabolomics techniques have been developed for foods such as olive oil, honey, milk, coffee, tea, saffron, and fruit juices to assess their geographic origin [31].

In this study, we compared the nutritional composition of Japanese Wagyu beef and other Wagyu beef bred in other countries using various MS techniques. We obtained the lipid profiles of Japanese Wagyu beef using high-performance liquid chromatography (HPLC)–MS/MS and also investigated the differences in fatty acid, triacylglyceride (TG), and trace-element profiles among Wagyu breeds. These differences in nutrients can be used to determine the production area of Wagyu cattle.

## 2. Results

### 2.1. Comparison of Single-Nucleotide Polymorphism Profiles in Cattle

**Figure 1:**
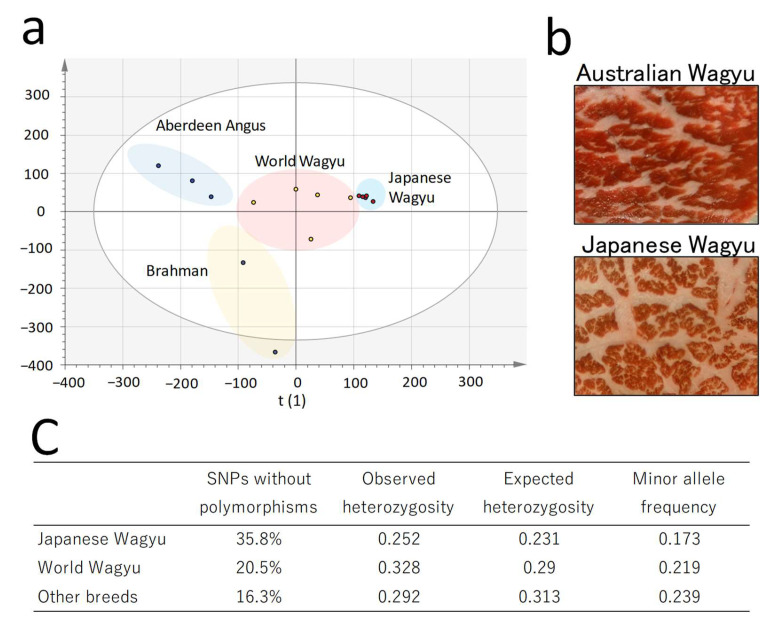
Genetic diversity map of global Wagyu cattle. The results of bovine single-nucleotide polymorphism arrays using 15 cattle, including Japanese Wagyu (five distinct producing regions in Japan), World Wagyu (one from USA, three from Australia, and one from New Zealand), and other breeds (three Aberdeen Angus and two tropical Brahman). (**a**) Score plot of principal component analysis detailing the relationship between Wagyu beef (scaling, UV; R2X (1) = 0.114; R2X (2) = 0.106). (**b**) Photograph of a cross-sectional view of the adductor muscle of Japanese and Australian Wagyu. (**c**) Comparison of genetic diversity among the Wagyu breeds. The table lists the results of each item in the PLINK whole-genome association analysis (each item n = 5).

We performed a genetic analysis of the Wagyu cattle from Japan and other countries using single-nucleotide polymorphism (SNP) arrays. Principal component analysis (PCA) of SNPs indicated the crossbreeding of Japanese Black cattle genes in the analyzed samples produced outside Japan (World Wagyu) (Figure 1a). In addition to Wagyu cattle, we performed genetic analyses of the Aberdeen Angus [32] and tropical Brahman breeds [33]. These breeds are considered to have been crossbred with World Wagyu breeds. SNPs in five Japanese Black cattle displayed a high degree of genetic similarity. A cross-sectional observation of beef suggested that marbling, in which adipocytes intermingle with muscle, is less common in World Wagyu than in Japanese Wagyu (Figure 1b). The texture of beef after cooking also confirmed the differences in the quality of beef from Wagyu breeds (Figure S1).

The heterozygosity values were indicative of genetic polymorphism between Japanese and World Wagyu beefs (Figure 1c). Compared with Japanese Wagyu, the percentage of interbred Wagyu genes in World Wagyu crossbreeds was estimated at 50–75% [26]. 

### 2.2. Characterization of TG Molecular Species in Wagyu

We focused on the lipid molecules in the meat to distinguish the geographic production area of Wagyu beef using methods other than genetic analysis. In a previous study, the total lipids fractionated via solvent extraction were comprehensively analyzed using LC–MS/MS (LTQ-Orbitrap XL, Thermo Fisher Scientific, Waltham, MA, USA), and 108 lipid molecules were identified [34]. Thus, we analyzed the total lipids in Wagyu beef using a more sensitive quadrupole-Orbitrap LC–MS/MS system (Q Exactive Plus).

Fourteen lipid classes were detected from the total lipid fraction extracted from the longissimus thoracis of Japanese Wagyu cattle (Table 1a). TGs were most abundant in Japanese Wagyu beef, and 128 TG molecular species with different combinations of fatty acids were identified. This excluded positional isomers because LC–MS/MS cannot analyze stereospecificity. The top 20 most abundant molecular species of TGs in Japanese Wagyu beef are listed in Table 1b. Following TGs, other abundant lipid molecular species included phosphatidylcholine (PC), lysophosphatidylcholine (LPC), diglycerides (DGs), and lysophosphatidylethanolamine (LPE). The top 10 lipid molecular species detected in these classes are presented in Figure S2.

Under these LC–MS/MS conditions, 259 lipid molecular species were identified, which exceeded the number reported previously [34]. Among the lipid molecules detected in Wagyu beef, TGs accounted for >70% of the total lipids. Furthermore, the top 15 molecular species of TGs accounted for >80% of the total TGs. Most lipids in beef comprised the major TGs. In other words, the lipids in Wagyu beef comprised several major types of TGs.

Fingerprinting with many lipid molecular species is highly accurate in discriminating Wagyu breeds. However, the use of LC–MS/MS is limited by its high cost. Therefore, we performed a comparative analysis of TGs in Wagyu beef using a conventional HPLC method [34].

Japanese, Australian, and Hybrid Wagyu beef were used as samples to distinguish between the geographic production areas of Wagyu beef. Hybrid Wagyu is a crossbreed between Angus and Wagyu breeds in Australia, and the calves of this breed are fattened for 16 months in Japan. The HPLC of TGs in the marbling of Wagyu beef revealed 16 TG fractions.

**Figure 2:**
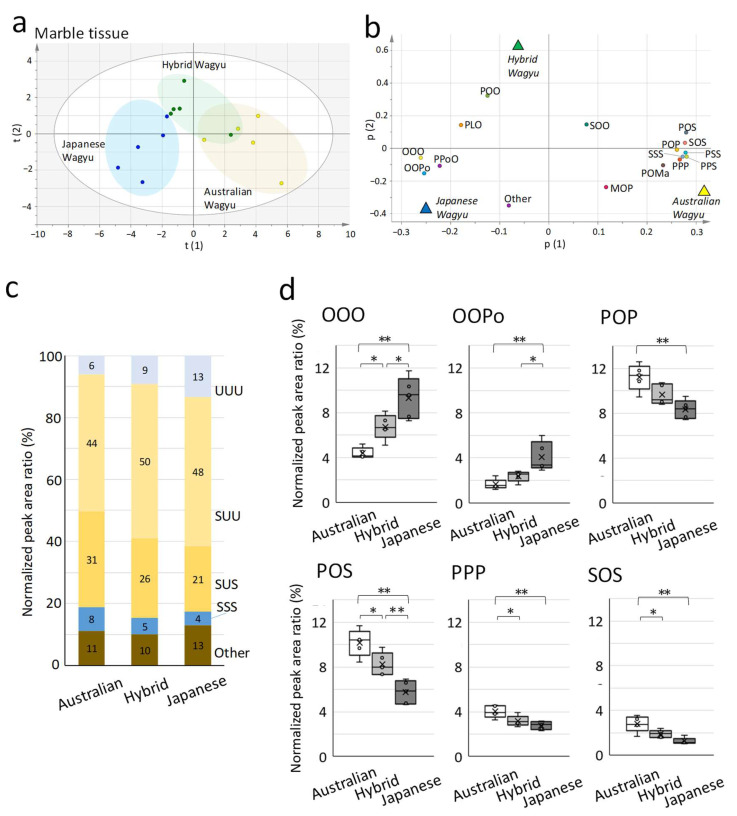
Comparison of triacylglycerides (TG) molecular species among the Wagyu breeds. We used high-performance liquid chromatography to determine the major TGs in Wagyu beef. Marbling tissues from the rib-eye area of the longissimus thoracis muscle (five Australian, five Hybrid, and five Japanese Wagyu) were used. (**a**) The score plot of orthogonal part least-square discrimination analysis (OPLS-DA) illustrated the relationship among Wagyu beef types (scaling, UV). (**b**) Loading plot detailing the relationship between TGs and each Wagyu type (R2X (1) = 0.606; R2X (2) = 0.152). Italicized letters indicate the location of each DA plot (triangles) obtained by OPLS-DA. (**c**) Percentage of unsaturated fatty acids in TGs. Bar graphs present the TG composition of each breed (each Wagyu, n = 5). The three-letter designations indicate the percentage of unsaturated and saturated fatty acids in the TGs. (**d**) Box plot of TGs presenting the exclusive median and all plots, including outliers. The cross marks indicate the mean (n = 5). Significant differences are presented as follows: * *p* < 0.05 and ** *p* < 0.01 (Tukey’s test; Australian vs. Hybrid vs. Japanese Wagyu). Abbreviations: U, unsaturated fatty acids; S, saturated fatty acids.

Orthogonal least-square discriminant analysis (OPLS-DA) score plots demonstrated a clear left–right separation of scores for the Japanese and Australian Wagyu (Figure 2a). The loading plots revealed a correlation between TG molecular species and the Wagyu breed (Figure 2b). The ratio of unsaturated fatty acids in TGs detected by HPLC is presented in Figure 2c. Japanese Wagyu featured more TGs (UUU), which contain three unsaturated fatty acids (abbreviated as U), than Australian and Hybrid Wagyu beef.

On comparing the molecular species of TGs, Japanese Wagyu beef featured significantly more triolein (OOO), which comprised three oleic acids (C18:1, abbreviation: O), and OOPo, which comprised two oleic acids and palmitoleic acid (C18:1; Po), than Australian and Hybrid Wagyu beef (Figure 2d). Moreover, Australian and Hybrid Wagyu beef contained significantly more POP and PPP, including palmitic acid (C16:0; P), and POS and SOS, including stearic acid (C18:0; S), than Japanese Wagyu beef.

**Figure 3:**
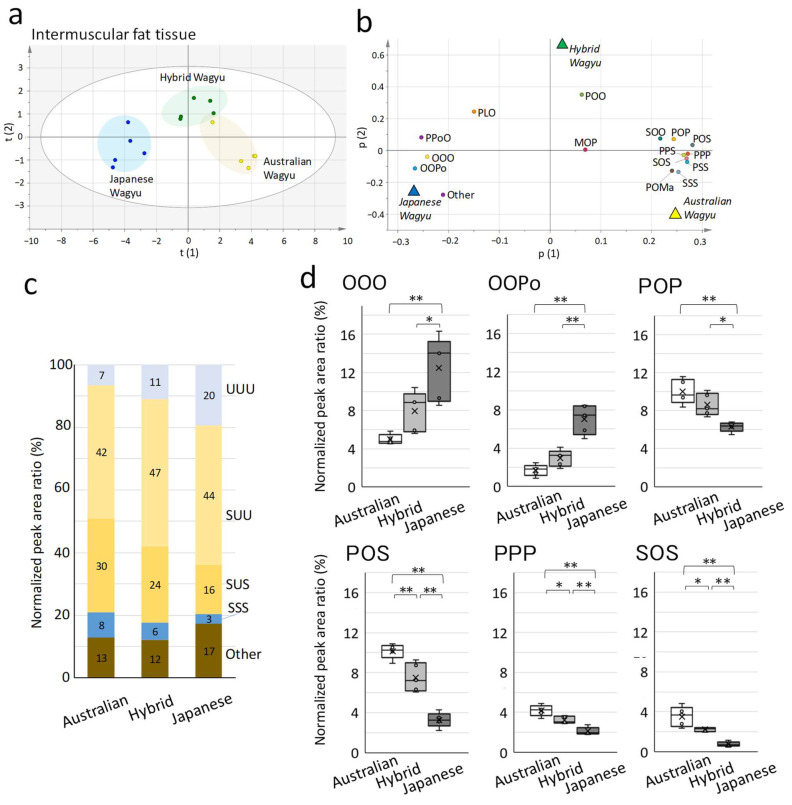
Comparison of triacylglycerides (TG) molecular species in intermuscular fat among Wagyu breeds. We used conventional high-performance liquid chromatography to determine the major TGs in Wagyu beef. Intermuscular fat tissues from the rib-eye area of the longissimus thoracis muscle (five Australian, five Hybrid, and five Japanese Wagyu) were used. (**a**) The score plot of orthogonal part least-square discrimination analysis (OPLS-DA) presents the relationship among the Wagyu beef types (scaling, UV). (**b**) Loading plot detailing the relationship between TG and each Wagyu breed (R2X (1) = 0.660; R2X (2) = 0.072). Italicized letters indicate the location of each DA plot (triangles) obtained by OPLS-DA. (**c**) Percentage of unsaturated fatty acids in TGs. Bar graphs present the fatty acid composition (each Wagyu, n = 5). The three-letter designations indicate the percentage of unsaturated and saturated fatty acids in the TGs. (**d**) Box plot of the exclusive median and all plots, including outliers. The cross marks indicate the mean (n = 5). Significant differences are indicated as follows: * *p* < 0.05 and ** *p* < 0.01 (Tukey’s test; Australian vs. Hybrid vs. Japanese Wagyu). Abbreviations: U, unsaturated fatty acids; S, saturated fatty acids.

Next, we compared the TGs in the fat tissue from Japanese, Australian, and Hybrid Wagyu cattle. Fat tissue samples were collected from intermuscular fat around the rib-eye of the longissimus thoracis muscle [34]. The TG compositions of intermuscular fat and marbling were similar among all the Wagyu breeds (Figure 3a,b). Analysis of the intermuscular fat of the Wagyu breeds demonstrated that POO, POS, POP, SOO, and PPoO were the major TGs in Australian Wagyu, whereas POO, OOO, PPoO, POP, and SOO were dominant in Japanese Wagyu. Hybrid Wagyu exhibited an intermediate TG ratio between Australian and Japanese Wagyu. TGs (UUU) accounted for 20% of the intermuscular fat and 14% of the marbling (Figure 3c). The detailed compositions of TGs and fatty acids in the marbling and intermuscular fat of each Wagyu breed are listed in Table 2 and Table 3.

Comparing the fatty acids in the marbling (Table 2), Japanese, Hybrid, and Australian Wagyu contained 50.3 ± 2.6, 47.1 ± 2.3, and 40.8 ± 2.8% oleic acid, respectively, and 4.9 ± 0.8, 3.8 ± 0.6, and 3.3 ± 0.8% palmitoleic acid, respectively, as monounsaturated fatty acids. However, considering the saturated fatty acids in marbling, Japanese, Hybrid, and Australian Wagyu contained 22.2 ± 2.3, 24.3 ± 1.1 and 26.0 ± 1.2% palmitic acid, respectively, and 8.0 ± 1.2, 10.3 ± 1.0, and 13.6 ± 2.6% stearic acid, respectively.

Comparing the fatty acid composition of the marbling and intermuscular fat from various Wagyu breeds, we found that oleic acid, palmitoleic acid, and myristoleic acid (C14:1) were more abundant in the intermuscular fat (Table 3).

### 2.3. Comparison of the Composition of Elements and Metabolites in Wagyu Beef

Elemental analysis using a mass spectrometer is used to distinguish the geographic production areas of meat [35,36]. In this study, we conducted an elemental analysis to compare the proportions of 24 elements in Japanese, Hybrid, and Australian Wagyu breeds. Among the 24 elements, four (nickel, arsenic, lanthanum, and cerium) were excluded from the data analysis because of their high variability.

**Figure 4:**
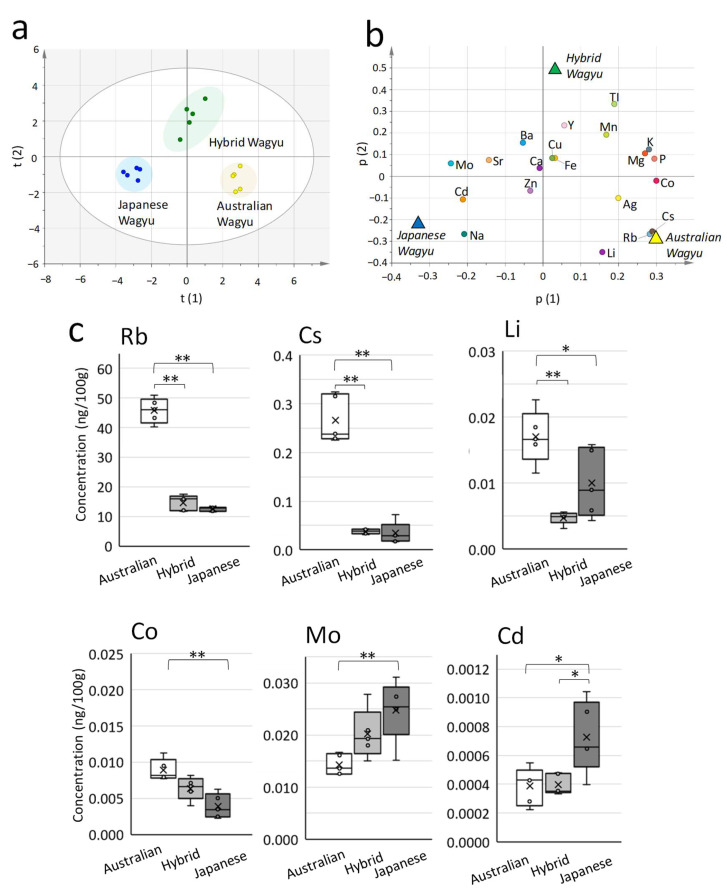
Comparison of Wagyu beef by elemental analysis. ICP–OES and ICP–MS were used to detect elements in beef. Samples were obtained from the rib-eye areas of the longissimus thoracis muscle (five Australian, Hybrid, and Japanese Wagyu each). (**a**) The score plot of orthogonal least-square discriminant analysis (OPLS-DA) reveals the relationships among Wagyu beef types regarding the musculus longissimus (scaling, UV). (**b**) Loading plot presenting the relationship between elements and each Wagyu type (R2X (1) = 0.309; R2X (2) = 0.149). Italicized letters indicate the location of each DA plot (triangles) obtained by OPLS-DA. (**c**) Box plot of the exclusive median and all plots, including outliers. The cross marks indicate the mean (n = 5). Significant differences are presented as follows: * *p* < 0.05 and ** *p* < 0.01 (Tukey’s test; Australian vs. Hybrid vs. Japanese Wagyu). Abbreviations: silver (Ag), barium (Ba), calcium (Ca), cadmium (Cd), cobalt (Co), cesium (Cs), bronze (Cu), iron (Fe), potassium (K), lithium (Li), magnesium (Mg), manganese (Mn), molybdenum (Mo), sodium (Na), phosphorus (P), rubidium (Rb), strontium (Sr), titanium (TI), yttrium (Y), zinc (Zn).

The score plot of OPLS-DA with 20 elements (Figure 4a) reveals a left–right split between Japanese and Australian Wagyu and an up–down split between Hybrid and Japanese Wagyu. The loading plot presents the correlation between elements and Wagyu breeds (Figure 4b). Rubidium (*p* < 0.001), cesium (*p* < 0.001), cobalt (*p* < 0.001), and lithium (*p* < 0.05) were present at significantly higher levels in Australian Wagyu beef than in Japanese Wagyu (Figure 4c). Rubidium (*p* < 0.001), cesium (*p* < 0.001), and lithium (*p* < 0.05) were also present at significantly higher levels in Australian Wagyu beef than in Hybrid Wagyu beef.

However, molybdenum (*p* < 0.01), and cadmium (*p* < 0.05) were present at higher levels in Japanese Wagyu beef. No elements were present at significantly higher levels in Hybrid Wagyu than in Japanese and Australian Wagyu beef.

**Figure 5:**
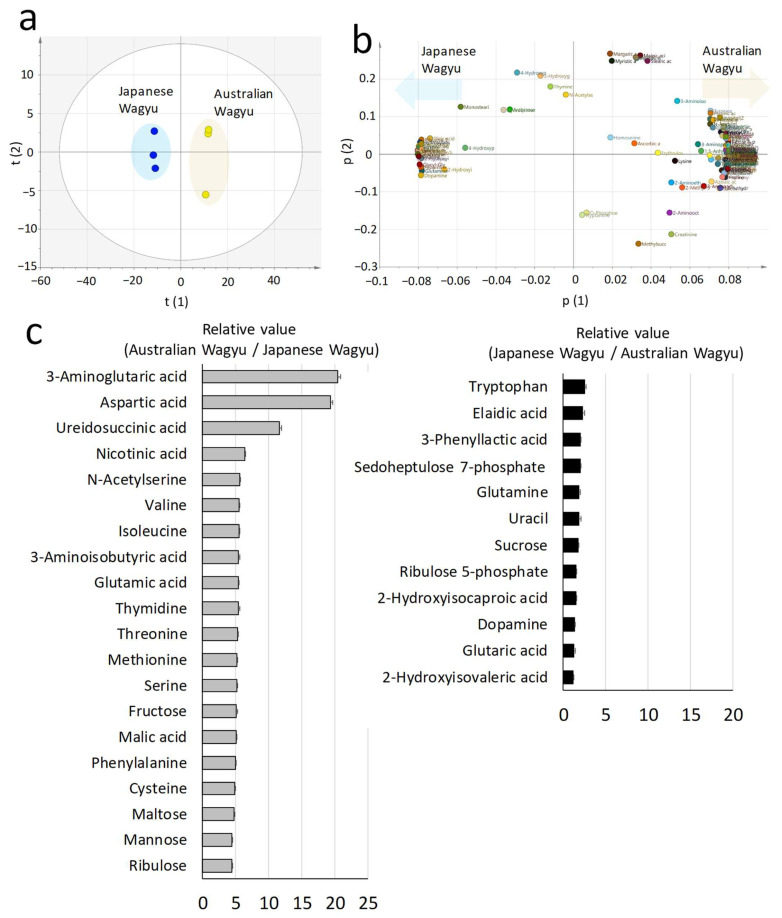
Comparison by metabolomics analysis. The figure presents the principal component analysis (PCA) results of the nutrients detected during the metabolomics analysis of the longissimus thoracis muscle from Japanese and Australian Wagyu. (**a**) Score plot of the PCA results for the metabolites of Japanese and Australian Wagyu beef (scaling, UV). (**b**) Loading plots presenting the relationship between metabolites and each Wagyu type (R2X (1) = 0.851; R2X (2) = 0.0623). Italicized letters indicate the location of each DA plot (triangles) obtained by orthogonal least-square discriminant analysis. The left and right sides of the circle indicate the correlations of metabolites with Japanese and Australian Wagyu, respectively. (**c**) Metabolite characteristics of Japanese and Australian Wagyu. The mean relative values of metabolites with significant differences (*p* < 0.01, n = 3, *t*-test) in the comparison of Wagyu cattle are presented in the graph. The graphs show the mean ± SD of relative values for Australian/Japanese Wagyu (left) and Japanese/Australian Wagyu (right).

Various metabolites, such as organic and amino acids, are used to discriminate Wagyu breeds [30,37]. Therefore, we used triple-quadrupole GC–MS to compare 475 metabolites in Japanese and Australian Wagyu beef. Under the same conditions, 167 metabolites were detected using GCMS-TQ8030. The score plot of PCA with 160 metabolites displays presents a left–right split for the Japanese and Australian Wagyu breeds (Figure 5a). The loading plots reveal the correlations between metabolites and Wagyu breeds (Figure 5b). Australian Wagyu beef contained more metabolites at higher levels than Japanese Wagyu beef. Japanese Wagyu beef featured higher levels of 23 metabolites than Australian Wagyu, and Australian Wagyu beef contained higher levels of 117 metabolites than Japanese Wagyu. The top 20 metabolites with greater abundance in Australian Wagyu with significant differences (*p* < 0.01) and coefficients of variation (<20%) are presented in Figure 5c. Among the 23 metabolites more abundant in Japanese Wagyu, 12 metabolites with significant differences (*p* < 0.01) and coefficients of variation (<20%) are also presented. The overview of the metabolite sets enriched in Kyoto Encyclopedia of Genes and Genomes (KEGG) analysis is presented in Figure S4.

## 3. Discussion

Various cattle breeds are produced globally, and their flavor profiles have been studied [34,38,39]. Wagyu cattle have traditionally been produced in Japan. There are four Japanese Wagyu breeds: Japanese Black, Japanese Brown, Japanese Polled, and Japanese Shorthorn [4]. Because the Japanese Black breed accounts for >97% of Japan’s Wagyu production [18], we referred to it as Japanese Wagyu in this study.

Japanese Wagyu is known to have low rates of genetic polymorphism because of its closed breeding system [26]. World Wagyu breeds include Australia, New Zealand, and USA varieties; this makes it difficult to estimate the degree of Wagyu crossbreeding using heterozygosity values. However, the percentage of Wagyu genes is estimated at 50%–75% based on the distribution of PCA data.

Japanese Wagyu has a characteristic lipid profile with high oleic acid content [40]. In this analysis, we used a high-resolution hybrid quadrupole-Orbitrap LC–MS/MS system to identify more Wagyu lipid molecules than those reported in previous studies [34]. Thus, we isolated and quantified 255 lipid molecules comprising major and rare lipids. The improved resolution of MS allowed the detection of rare TG molecules, including the odd-chain fatty acids margaric acid (C17:0) and heptadecenoic acid (C17:1). These fatty acids are derived from ruminant grass fermentation [41]. The major TGs (e.g., POO, POS, POP, SOO, and PLO) detected by LC–MS/MS were consistent with previous data [34]. However, detailed comparisons of the lipid molecules of cattle breeds or livestock using MS require careful consideration of the coefficient of variation of the data and differences in MS devices [42,43].

Using conventional HPLC methods, the comparison of TGs in Japanese, Hybrid, and Australian Wagyu beef revealed significant differences in OOO, OOPo, POP, POS, PPP, and SOS content. The expression of lipid-metabolizing enzymes that synthesize unsaturated fatty acids, such as acetyl-CoA acetyltransferase and the elongation of very long-chain fatty acids protein, is significantly upregulated in Japanese Black cattle compared with that in other breeds [44]. The differences in TG composition between Japanese and Australian Wagyu beef are suggested to reflect the different degrees of genetic crossbreeding in the corresponding countries. In addition, a comparison of Hybrid and Australian Wagyu beef suggests that the TG composition reflects the climate and feed composition of each country.

We assume that the PCA data of marbling were less tightly clustered than those of intermuscular fat because of the variability of fat–muscle intermingling. The comparison of OOO containing three oleic acids among Japanese, Hybrid, and Australian Wagyu revealed more significant differences in intermuscular fat than in marbling tissue. TGs in Wagyu beef feature high levels of oleic acid. Oleic acid is an omega-9 fatty acid with the physiological function of having anti-inflammatory effects [45]. Oleic acid is a partially essential fatty acid that must be acquired from diet. Oleic acid has also been reported to have antitumor effects. Moderate consumption of Wagyu beef, which is high in oleic acid, may have health benefits [46].

ICP–MS detects trace elements (detection sensitivity: pg/g to ng/g) by analyzing ionized elements, but major elements present at high levels are detected by ICP–OES, thus reducing blind spots in the nutritional components and improving evaluation accuracy (Figure S5). A comparative elemental analysis detected significantly higher cadmium and molybdenum levels in Japanese Wagyu beef. One reason for this might be that Japanese soils contain higher cadmium and molybdenum levels than soils in other countries [47]. Significantly higher levels of cesium, rubidium, and lithium, which are alkali metals, were detected in Australian Wagyu beef. Moreover, the alkali metal sodium was detected at higher levels in Japanese Wagyu beef. Alkali metals are present in trace amounts in water, soil, and feed, and they are absorbed in the body with similar behavior. The profiles of alkali metals suggest a clear difference in the Japanese and Australian Wagyu cattle production areas.

A comparison of Japanese and Australian Wagyu beef by metabolomics analysis demonstrated that many amino acids derived from lean meat are present in Australian Wagyu beef. Amino acid metabolism and biosynthesis were the top enriched metabolite sets in KEGG analysis (Figure S4). This result is similar to the comparison between the dairy cattle breeds Holstein and Japanese Wagyu [48].

Further, elaidic acid, sedoheptulose 7-phosphate, ribose 5-phosphate, and other amino acids were present at higher levels in Japanese Wagyu beef. Elaidic acid is a transisomer of oleic acid. In a previous study, we reported that elaidic acid is a metabolic marker of intermuscular fat [37]. Sedoheptulose 7-phosphate and ribose 5-phosphate are intermediate metabolites in the pentose phosphorylation pathway. This pathway is involved in the biosynthesis of nucleic acids and is enhanced in Wagyu cattle [37,48].

**Figure 6:**
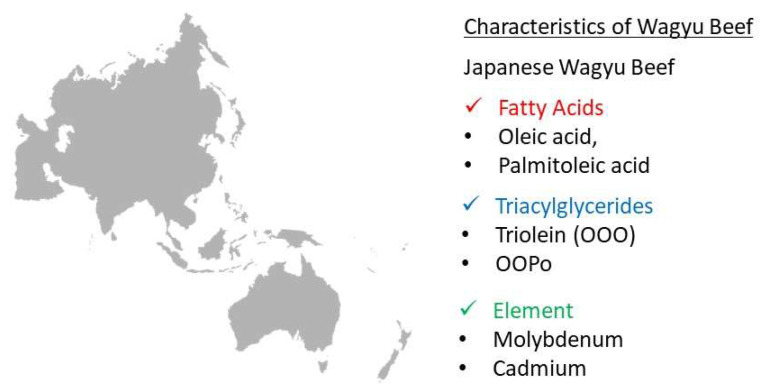
Characteristics of Wagyu breed determined via comparative analysis. A summary of the distinct components of Wagyu was obtained by lipid and elemental analyses.

Conversely, metabolite changes during aging after slaughter have been identified in beef via metabolomic analysis [49]. Therefore, it is possible that many of the metabolites detected in Australian Wagyu beef may be attributable to proteolysis during postmortem aging. When conducting metabolomic analysis of metabolite changes, one should consider the conditions and duration of Wagyu beef storage (Figure 6). Further studies on Wagyu cattle are warranted to increase the number of samples.

## 4. Materials and Methods

### 4.1. Sample Collection

The longissimus thoracic or adductor muscles of Japanese Black cattle (production area: multiple prefectures in Japan) or Hybrid Wagyu (a cross between Angus and Wagyu born in Australia, transported to Japan as calves, and grown for 16 months in Japan) were commercially purchased from livestock farmers in Japan. The longissimus thoracic or adductor muscle of World Wagyu (production area: Australia, New Zealand, and USA) was commercially purchased through a trading company. No animal experiments were included in this study.

### 4.2. SNP Analysis Using the Illumina Bovine Chip

Genomic purification from tissues was performed using the phenol–chloroform method. Purity was checked using a NanoDrop One Spectrophotometer (Thermo Fisher Scientific). SNP genotyping was conducted using Illumina Bovine SNP50K BeadChip ver. 3.0 (Illumina K.K., Tokyo, Japan). Of the SNPs in the chip, SNPs located on autosomal chromosomes were used in the analysis, excluding the SNPs on unknown or sex chromosomes. The observed heterozygosity, expected heterozygosity, and minor allele frequencies were calculated using 50,868 SNPs, excluding 861 SNPs for which genotyping was unsuccessful in the sample. After quality control, PCA was performed using 44,546 SNPs with a SNP call rate of ≤95%, minor allele frequency of ≤5%, and a Hardy–Weinberg equilibrium of ≤0.001. The SNP data were analyzed using PLINK v.1.9 (http://pngu.mgh.harvard.edu/purcell/plink/ accessed on 13 January 2020) [50].

### 4.3. LC–MS/MS Analysis of TGs

The total lipid fraction was extracted from 1 mg of the Bligh and Dyer variant sample. The extract was dried with nitrogen gas, redissolved in methanol, and subjected to LC–MS/MS. LC–MS/MS was performed using DIONEX UtiMate 3000 (Thermo Fisher Scientific) with an L-column3 C18 metal-free column (2 mm inner diameter, 100 mm length, and 2 μm particle size; Chemicals Evaluation and Research Institute, Tokyo, Japan) and Q Exactive Plus (Thermo Fisher Scientific). The HPLC system comprised solvents A (5 mM ammonium formate solution, 0.05% ammonium hydroxide in the solvent mixture [2-propanol:methanol:water = 5:1:4]) and B (5 mM ammonium formate, 0.05% ammonium hydroxide, 2-propanol). The HPLC conditions included a column temperature of 40 °C, an injection volume of 10 μL, and a flow rate of 0.1 mL/min. MS was performed using the Full MS/dd-MS2 mode (TopN:20) from 200 *m*/*z* to 1800 *m*/*z*. MS of TGs was performed in the positive ion mode. The internal standards were TG (15:0/18:0/15:0) *d_5_* for TG, PC (18:1*d_7_*/15:0) for LPC and PC, phosphatidylethanolamine (PE, 18:1*d_7_*/15:0) for PE and LPE, DG (17:0/17:0) *d_5_* for DG, phosphatidylserine (PS, 18:1*d_7_*/15:0) for PS, phosphatidylinositol (PI, 18:1*d_7_*/15:0) for lysophosphatidylinositol and PI, monoglyceride (MG, 18:1*d_7_*) for MG, phosphatidylglycerol (PG, 18:1*d_7_*/15:0) for PG, cardiolipin (CL, 14:0/14:0/14:0/14:0) for CL, sphingomyelin (SM, _d_18:1/12:0) for SM, and ceramide (Cer, _d_18:1/12:0) for Cer. These standards were purchased from Avanti Polar Lipids, Inc. (Alabaster, AL, USA). The TG molecular species were identified using Lipid Search v.4.2.23 (Mitsui Knowledge Industry Co., Ltd., Tokyo, Japan) lipid-identification-analysis software.

### 4.4. Analysis of the Fatty Acid Composition of TGs

Total lipids were extracted from ground beef (10 g) with *t*-butyl methyl ether/methanol (2:1) following a previously described method [34]. The fatty acid composition of TGs was determined by GC (GC-2010 Plus; Shimadzu, Kyoto, Japan) using a TC-70 capillary column (60 m length, 0.25 mm i.d., and 0.25 μm film thickness; GL Sciences) after methyl esterification according to conventional methods [34].

For the TG analysis, samples dissolved in isopropyl alcohol were subjected to HPLC using an Agilent Technologies 1260 Infinity equipped with a refractive index detector and Poroshell 120 EC-C18 LC column (three columns in a series: 3.0 mm × 50 mm, 3.0 mm × 50 mm, and 3.0 mm × 100 mm; 2.7-Micron; Agilent Technologies, Santa Clara, CA, USA) following a previously described method [34]. The quantification of individual TG species was performed by evaluating the corresponding relative percentage according to the normalization area procedure.

### 4.5. Multielement Analysis by ICP–MS and ICP–OES

A ceramic knife (#FKR-180CX-FP, Kyocera, Kyoto, Japan) was used to grind 50 g samples. The ground samples were then lyophilized in a freeze-dryer (#DRZ350WA, Advantech, Tokyo, Japan). Each dried sample was crushed finely (to <3 mm) with a plastic hammer.

Defatted samples were prepared by removing the lipid using organic solvent (hexane and 2-propanol 3:2) and washed with hexane.

A 20-fold volume of 61% nitric acid (high-purity electronics, industrial grade, Kanto Chemical, Tokyo, Japan) was added to the defatted samples, which were heated at 120 °C on an electric hot plate (#EC-1200N, Asone, Osaka, Japan). Next, after nitric acid digestion, 70% perchloric acid (TAMAPURE-AA-100, Tama Chemicals, Kanagawa, Japan) was added to the solution. The samples were then heated at 200 °C until the organic compounds in the solution were completely digested. The solvent was evaporated by heating, and 1% nitric acid was added to the dried sample to completely dissolve the residue. An internal standard (5 µg/L) was added to the sample. The sample solution was used for the elemental concentration measurement after constant volume addition with 1% nitric acid. All samples were analyzed in duplicate.

We used different analytical instruments depending on the element characteristics. The elements were divided into two groups according to their properties. The first group comprised Li, Co, Cu, Rb, Y, Mo, Ag, Cd, Cs, and Ti, which were analyzed by ICP–MS (Varian 820MS, Agilent Technologies). The second group comprised Na, Mg, P, K, Ca, Mn, Fe, Zn, Sr, and Ba, which were analyzed by ICP–OES (Varian 725-ES, Agilent Technologies). A summary of the analyzer conditions and elemental references is presented in Figure S5.

### 4.6. Metabolomics Analysis by GC–MS

Here, 1 mL of methanol containing an internal standard (2-isopropylmalic acid) was added to 100 mg of tissue crushed with zirconia beads (Tissue Lyser, QIAGEN K.K., Tokyo, Japan). After centrifugation, 100 μL each of ultrapure water and chloroform were added to 250 μL of supernatant and shaken at 1200 rpm for 5 min at 37 °C using a thermomixer comfort shaker (Eppendorf, Hamburg, Germany). Subsequently, 250 μL of ultrapure water were added to this solution and centrifuged at 16,000× *g* for 10 min at 4 °C to collect the supernatant as the water-soluble fraction of the chloroform/methanol extraction. The water-soluble extract was completely dried via centrifugal drying (#CC-105, Tomy Seiko, Tokyo, Japan) for 40 min and freeze-drying for 16 h. The dried samples were dissolved in pyridine containing 20 mg/mL methoxyamine hydrochloride for 90 min at 37 °C with shaking at 1200 rpm at 37 °C. The mixture was then derivatized by shaking with N-methyl-N-trimethylsilyl trifluoroacetamide (GL Sciences) for 30 min at 1200 rpm at 37 °C. After centrifugation, the supernatant was used for GC–MS and processed on a GCMS-TQ8030 (Shimadzu Co.) with a BPX-5 capillary column (30 m × 0.25 mm; 0.25 μm film thickness, Trajan Scientific Japan, Kanagawa, Japan). The column temperature was maintained at 60 °C for 2 min, increased by 15 °C/min to 330 °C, and then maintained at 330 °C for 3 min. The front inlet temperature was maintained at 280 °C. The helium gas flow rate was 39.0 cm/s. The interface and ion-source temperatures were 280 and 200 °C, respectively. The analysis mode was set to selected reaction monitoring with a split ratio of 1/30 and ionization voltage of 70 V. The mass spectrum was analyzed using GC/MS Metabolite Database v.2 (Shimadzu).

### 4.7. Validated Methods for GC–MS and LC–MS/MS

A mixture of all samples was used for quality control, and only the molecular species with high confidence in the measured data’s peak shape, intensity, and elution time were quantified. Analyzed samples were measured by randomizing their order. The height of the detected peak in each sample was corrected by an internal standard and sample weight. The solvent blank value was subtracted, and those with negative values were treated as nondetects.

### 4.8. Statistical Analyses

Statistical significance was determined using Student’s *t*-test with Excel 2019 (Microsoft Japan, Tokyo, Japan) or Tukey’s method with Bellcurve (Social Survey Research Information, Tokyo, Japan). Statistical processing, such as log transformation and scaling, and multivariate analysis (OPLS-DA and PCA) were performed using SIMCA 14.1 software (Infocom, Tokyo, Japan) [8,34].

## 5. Conclusions

To distinguish between the geographic origins of Wagyu beef via omics analysis, we compared the differences in the nutritional composition of Wagyu beef from different countries using MS. Quadrupole-Orbitrap LC–MS/MS systems were used to detect 259 lipid molecules, including TG, PC, LPC, DG, and LPE. Because the significant TGs dominate the lipid profile of Wagyu beef, we compared the TG content of Japanese, Hybrid, and Australian Wagyu beef using conventional HPLC. OOO and OOPo were significantly more abundant in Japanese Wagyu beef, whereas POP, POS, PPP, and SOS were significantly more abundant in Australian Wagyu beef. Next, elemental comparisons based on ionomics analysis using ICP–MS and ICP–OES demonstrated that Japanese Wagyu beef contained higher levels of the Mo and Cd trace elements, whereas Australian Wagyu beef contained significantly more Rb, Cs, and Li. Comparing metabolites via metabolomics analysis using GC–MS illustrated that Australian Wagyu beef contained significantly more amino acids and other components derived from lean muscle than Japanese Wagyu beef. These results of omics analyses revealed that the nutritional composition of Wagyu cattle differed with the country of production. This study suggests that combining multiple omics analyses enables the discrimination of the origin of genetically similar Wagyu cattle.

## Figures and Tables

**Table 1:** Comprehensive analysis of the lipid molecular species of Japanese Wagyu beef. The lipid composition was analyzed using a high-resolution hybrid quadrupole-Orbitrap LC–MS/MS system. The sample was the marbling tissue from the longissimus thoracis muscle (0 days after slaughter). (**a**) Mean ± standard deviation (SD) for each molecular species of lipid classified as simple and complex lipids (Japanese Wagyu, n = 5). (**b**) Top 20 triacylglycerides detected in Japanese Wagyu beef (Japanese Wagyu n = 5).

(a) Total Amount of Each Lipid	Abbreviation	Mean ± SD (pmol/mg)	Number of Detections
Triglyceride	TG	18,797 ± 7,753	128
Phosphatidylcholine	PC	3,216 ± 590.3	31
Lysophosphatidylcholine	LPC	1,356 ± 360.3	10
Diglyceride	DG	1,205 ± 2023.2	25
Lysophosphatidylethanolamine	LPE	629 ± 175.6	10
Phosphatidylethanolamine	PE	513 ± 200.4	25
Phosphatidylinositol	PI	301 ± 34.4	9
Phosphatidylserine	PS	101 ± 7.6	4
Sphingomyeline	SM	91 ± 25.7	4
Monoglyceride	MG	77 ± 72.9	4
Cardiolipin	CL	74 ± 23	2
Ceramides	Cer	16 ± 1.6	5
Lysophosphatidylinositol	LPI	13 ± 4.6	1
Phosphatidylglycerol	PG	7 ± 1.6	1
**(b) Triacylglyceride**	**Symbol**	**Mean ± SD (pmol/mg)**	**% of all TGs**
TG(C16:0/C18:1/C18:1)	POO	3,471 ± 1231	18.5
TG(C16:0/C16:1/C18:1)	PPoO	1,564 ± 586.4	8.3
TG(C18:0/C16:0/C18:1)	POS	1,347 ± 602.9	7.2
TG(C16:0/C16:0/C18:1)	POP	1,259 ± 524.2	6.7
TG(C18:0/C18:1/C18:1)	SOO	1,183 ± 549.4	6.3
TG(C16:0/C18:1/C18:2)	PLO	1,086 ± 489	5.8
TG(C16:1/C18:1/C18:1)	OOPo	1,086 ± 488.7	5.8
TG(C16:0/C14:0/C18:1)	MOP	918 ± 415.4	4.9
TG(C18:1/C18:1/C18:1)	OOO	867 ± 452.7	4.6
TG(C16:0/C14:1/C18:1)	POMo	661 ± 289.7	3.5
TG(C16:0/C17:1/C18:1)	POHe	395 ± 192.7	2.1
TG(C18:0/C18:0/C18:1)	SOS	384 ± 242.4	2.0
TG(C17:0/C18:1/C18:1)	OOMa	364 ± 190.8	1.9
TG(C16:1/C16:1/C18:1)	OPoPo	284 ± 140.3	1.5
TG(C16:0/C16:1/C18:2)	PPoL	284 ± 139.9	1.5
TG(C18:0/C14:0/C16:0)	MOS	235 ± 148.1	1.3
TG(C16:0/C14:0/C16:1)	PPoM	231 ± 122.2	1.2
TG(C16:0/C17:0/C18:1)	POMa	226 ± 108.1	1.2
TG(C18:0/C16:0/C16:0)	PPS	217 ± 126.7	1.2
TG(C15:0/C16:0/C18:1)	OPPe	177 ± 76.7	0.9

**Table 2:** TGs and fatty acids in the marble tissue of each Wagyu sample.

Marble Tissue				
Triacylglycerides (%)		Australian Wagyu	Hybrids Wagyu	Japanese Wagyu
POO ^a^		27.8 ± 3.6	30.9 ± 1.9	28.9 ± 3.6
POP ^b^		11.2 ± 1.2	9.6 ± 0.9	8.3 ± 1.2
POS		10.2 ± 1.2	8.2 ± 1.0	5.7 ± 1.2
PPoO ^c^		7.3 ± 0.9	7.9 ± 1.0	8.8 ± 0.9
SOO		7.1 ± 0.7	7.2 ± 0.8	6.8 ± 0.7
MOP ^d^		5.1 ± 0.8	4.7 ± 0.3	4.6 ± 0.8
OOO		4.4 ± 0.5	6.7 ± 1.1	9.3 ± 0.5
PPP		4.0 ± 0.5	3.2 ± 0.5	2.8 ± 0.5
SOS		2.8 ± 0.7	1.9 ± 0.3	1.2 ± 0.7
PPS		2.3 ± 0.5	1.4 ± 0.3	1.1 ± 0.5
PLO		2.1 ± 0.3	3.9 ± 0.8	3.6 ± 0.3
OOPo		1.7 ± 0.5	2.4 ± 0.5	4.1 ± 0.5
POMa		1.5 ± 0.2	1.2 ± 0.3	1.1 ± 0.2
PSS		1.1 ± 0.4	0.7 ± 0.2	0.4 ± 0.4
SSS		0.3 ± 0.1	0.1 ± 0.1	0.0 ± 0.1
Other ^e^		11.1 ± 1.2	10.0 ± 1.6	13.0 ± 1.2
**Marble Tissue**				
**Fatty Acid (%)**	**Symbol**	**Australian Wagyu**	**Hybrids Wagyu**	**Japanese Wagyu**
C18:1	O	40.8 ± 2.8	47.1 ± 2.3	50.3 ± 2.6
C16:0	P	26.0 ± 1.2	24.3 ± 1.1	22.2 ± 2.3
C18:0	S	13.6 ± 2.6	10.3 ± 1.0	8.0 ± 1.2
C16:1	Po	3.3 ± 0.8	3.8 ± 0.6	4.9 ± 0.8
C18:2	L	2.7 ± 0.4	4.4 ± 1.0	4.2 ± 1.0
C17:0	Ma	1.0 ± 0.2	1.0 ± 0.2	0.7 ± 0.1
C14:1	Mo	0.9 ± 0.3	0.8 ± 0.2	1.3 ± 0.3
C15:0	Pe	0.4 ± 0.1	0.3 ± 0.1	0.3 ± 0.1
C18:3	Al	0.2 ± 0.0	0.1 ± 0.0	0.2 ± 0.0
C14:0	M	0.0 ± 0.0	0.0 ± 0.0	0.0 ± 0.0
Other ^e^	-	11.0 ± 1.1	7.7 ± 0.7	7.8 ± 1.5

Marbling from the musculus longissimus of Wagyu cattle (five Australian, Hybrid, and Japanese Wagyu each) were analyzed. Data represent the mean ± SD for each fatty acid and triacylglycerides (TG). The three capital letters in the table indicate ester-linked fatty acid combinations. They indicate the fatty acid compositions but do not reflect the positions of the glycerol backbone. The following TGs were presumed to be a mixture of TGs in parentheses with close chromatographic retention times: ^a^ POO (+SLO), ^b^ POP (+PLS), ^c^ PPoO (+MOO), and ^d^ MOP (+PLP). ^e^ Other indicates the sum of the peak areas of unidentified TG molecular species.

**Table 3:** TGs and fatty acids in the intermuscular fat of each Wagyu sample.

Intermuscular Fat				
Triacylglycerides (%)		Australian Wagyu	Hybrids Wagyu	Japanese Wagyu
POO ^a^		26.0 ± 2.3	27.7 ± 1.0	25.3 ± 1.3
POS ^b^		10.1 ± 0.7	7.5 ± 1.4	3.3 ± 0.7
POP		10.0 ± 1.3	8.6 ± 1.2	6.3 ± 0.5
SOO ^c^		8.1 ± 1.4	7.8 ± 0.6	5.5 ± 1.1
PPoO		6.3 ± 1.5	8.1 ± 0.2	10.2 ± 1.1
OOO ^d^		5.0 ± 0.5	8.0 ± 2.1	12.5 ± 3.4
MOP		4.7 ± 1.1	4.6 ± 0.4	4.3 ± 0.3
PPP		4.2 ± 0.6	3.2 ± 0.3	2.1 ± 0.4
SOS		3.5 ± 1.0	2.2 ± 0.2	0.8 ± 0.2
PPS		2.1 ± 0.5	1.4 ± 0.4	0.6 ± 0.1
PLO		2.0 ± 0.4	3.4 ± 0.8	3.5 ± 1.4
OOPo		1.7 ± 0.6	3.0 ± 0.9	7.0 ± 1.6
POMa		1.6 ± 0.2	1.3 ± 0.1	1.1 ± 0.2
PSS		1.3 ± 0.4	0.7 ± 0.2	0.2 ± 0.1
SSS		0.4 ± 0.2	0.2 ± 0.0	0.1 ± 0.1
Other ^e^		12.9 ± 1.3	12.1 ± 1.2	17.3 ± 2.5
**Intermuscular Fat**				
**Fatty Acid (%)**	**Symbol**	**Australian Wagyu**	**Hybrids Wagyu**	**Japanese Wagyu**
C18:1	O	40.9 ± 1.7	47.7 ± 3.2	52.4 ± 4.0
C16:0	P	23.9 ± 2.0	22.3 ± 2.4	18.6 ± 2.5
C18:0	S	15.4 ± 1.8	11.0 ± 1.1	5.6 ± 1.3
C16:1	Po	3.1 ± 0.9	4.2 ± 0.3	6.8 ± 1.0
C18:2	L	2.2 ± 0.3	3.6 ± 0.6	4.7 ± 1.1
C17:0	Ma	1.2 ± 0.2	1.0 ± 0.1	0.5 ± 0.1
C14:1	Mo	0.9 ± 0.3	1.1 ± 0.3	2 ± 0.6
C15:0	Pe	0.4 ± 0.1	0.4 ± 0.1	0.3 ± 0.1
C18:3	Al	0.2 ± 0.0	0.2 ± 0.0	0.2 ± 0.0
C14:0	M	0.0 ± 0.0	0.0 ± 0.0	0.0 ± 0.0
Other ^e^	-	11.7 ± 1.2	8.5 ± 0.9	8.7 ± 1.0

Intermuscular fat tissues of the musculus longissimus of Wagyu cattle (five Australian, Hybrid, and Japanese Wagyu each) were analyzed. The data are presented as the mean ± SD for each fatty acid and triacylglycerides (TG). The three capital letters in the table indicate ester-linked fatty acid combinations. They indicate the fatty acid compositions, but they do not reflect the positions of the glycerol backbone. The following TGs were presumed to be a mixture of TGs in parentheses with close chromatographic retention times: ^a^ POO (+SLO), ^b^ POP (+PLS), ^c^ PPoO (+MOO), and ^d^ MOP (+PLP). ^e^ Other indicates the sum of the peak areas of unidentified TG molecular species.

## Data Availability

The data presented in this study are available on request from the corresponding author. The data are not publicly available due to patent issues.

## Supplementary Materials

The following are available online at https://www.mdpi.com/article/10.3390/metabo12090777/s1, Figure S1: Comparison of beef hardness using a creep meter. Figure S2: Top 10 lipid molecular species of phosphatidylcholine, lysophosphatidylcholine, diglyceride, and lysophosphatidylethanolamine in Wagyu beef detected by LC–MS/MS. Figure S3: Comparison of sodium ions in Wagyu beef by elemental analysis. Figure S4. Results of Kyoto Encyclopedia of Genes and Genomes analysis of metabolomics data by GC–MS. Figure S5. Summary of analytical instrument conditions and elemental references for ICP–MS and ICP–OES.

## Abbreviations

LC–MS/MS	Liquid chromatography–tandem mass spectrometry
GC–MS	Gas chromatography–mass spectrometry
HPLC	High-performance liquid chromatography
SNP	Single-nucleotide polymorphism
OPLS-DA	Orthogonal part least squares discrimination analysis
PCA	Principal component analysis
SD	Standard deviation
TG	Triacylglyceride
DG	Diglyceride
MG	Monoglyceride
LPC	Lysophosphatidylcholine
LPE	Lysophosphatidylethanolamine
PC	Phosphatidylcholine
PE	Phosphatidylethanolamine
PI	Phosphatidylinositol
PS	Phosphatidylserine
PG	Phosphatidylglycerol
SM	Sphingomyelin
CL	Cardiolipin
Cer	Ceramide
