# Supplementary material for: Application of Mass Spectrometry for Determining the Geographic Production Area of Wagyu Beef
Source: Metabolites. 2022 Aug 23;12(9):777. doi: 10.3390/metabo12090777 (PMC9506216; doi:10.3390/metabo12090777)
Supplement: Supplementary file 1 [file metabolites-12-00777-s001.zip › metabolites-1800750-supplementary.pdf]

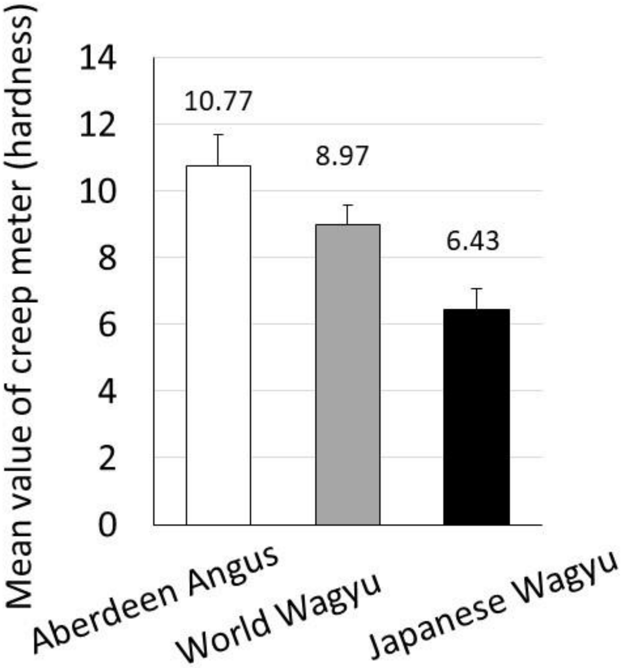

Figure S1. Comparison of beef hardness using a creep meter.

Beef (semimembranous muscle) was cut into 2-cm-thick pieces and cooked in hot water at 70°C for 20 min. Beef hardness was measured using a creep meter (Yamaden Co., Ltd.). The measurement conditions were as follows: the hardness was defined as the maximum load generated when the plunger (3-mm diameter cylinder) was inserted 15-mm deep into the meat.

Ascending speed of the specimen stand: 1 mm/s

Data represented as the mean ± standard deviation (n = 3).

	(pmol/mg)			
Phosphatidylcholine	Mean		SD	% of all PCs
PC(C16:0/C18:1)	1231.4	±	274.2	38.3
PC(C16:1/C18:1)	571.7	±	113.4	17.8
PC(C18:1/C18:1)	233.4	±	22.8	7.3
PC(C18:1/C18:2)	130.5	±	24.3	4.1
PC(C18:0/C18:1)	120.1	±	24.2	3.7
PC(C16:0/C16:1)	108.6	±	37.8	3.4
PC(C16:0/C20:4)	81.6	±	20.0	2.5
PC(C18:0/C17:0)	76.0	±	57.6	2.4
PC(C16:1e/C18:2)	73.8	±	50.6	2.3
PC(C16:1e/C20:3)	65.3	±	28.2	2.0

	(pmol/mg)			
Lysophosphatidylcholin	Mean		SD	% of all LPCs
LPC(C18:2)	499.9	±	162.8	36.9
LPC(C18:1)	463.2	±	196.7	34.2
LPC(C20:4)	171.8	±	35.4	12.7
LPC(C20:3)	70.6	±	14.2	5.2
LPC(C16:0)	37.3	±	19.4	2.8
LPC(C22:4)	32.6	±	12.1	2.4
LPC(C16:1)	25.7	±	14.8	1.9
LPC(C22:5)	23.4	±	4.8	1.7
LPC(C18:3)	21.6	±	4.1	1.6
LPC(C17:1)	10.1	±	6.4	0.7

	(pmol/mg)			
Diglyceride	Mean		SD	% of all DGs
DG(C16:0/C18:1)	403.1	±	692.7	33.46
DG(C18:1/C18:1)	301.8	±	563.5	25.06
DG(C16:1/C18:1)	100.6	±	152.5	8.35
DG(C18:0/C18:1)	97.7	±	174.6	8.11
DG(C18:1/C14:0)	88.7	±	137.3	7.36
DG(C16:0/C16:0)	40.8	±	63.5	3.39
DG(C18:1/C18:2)	32.9	±	53.4	2.73
DG(C16:0/C14:0)	22.7	±	30.5	1.88
DG(C16:0/C14:1)	21.6	±	27.2	1.79
DG(C18:1/C14:1)	19.7	±	29.7	1.64

	(pmol/mg)			
Lysophosphatidylethan	Mean		SD	% of all LPEs
LPE(C20:4)	204.0	±	31.4	32.4
LPE(C18:2)	172.1	±	59.8	27.4
LPE(C18:1)	94.2	±	57.5	15.0
LPE(C20:3)	60.9	±	13.7	9.7
LPE(C22:4)	45.4	±	18.6	7.2
LPE(C22:5)	29.7	±	4.7	4.7
LPE(C20:5)	7.5	±	1.0	1.2
LPE(C18:3)	5.7	±	1.5	0.9
LPE(C18:0)	4.7	±	2.4	0.8
LPE(C15:0)	4.6	±	1.5	0.7

Figure S2. Top 10 lipid molecular species of phosphatidylcholine, lysophosphatidylcholine, diglyceride, and lysophosphatidylethanolamine in Wagyu beef detected via liquid chromatography–tandem mass spectrometry.

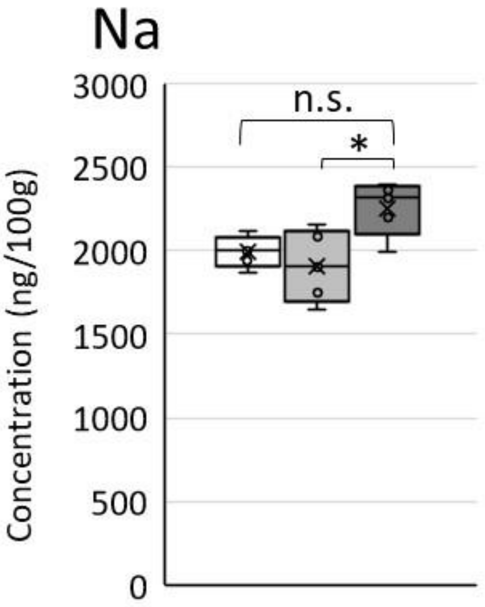

Figure S3. Comparison of sodium ions in Wagyu beef by elemental analysis. Inductively coupled plasma optical emission spectrometry and mass spectroscopy were used to detect elements in beef.
Samples were obtained from rib-eye areas of the longissimus thoracis muscle (five Australian, Hybrid, and Japanese Wagyu each). The box plot presents the exclusive median and all plots, including outliers. The cross marks indicate the mean (n = 5). Significant differences are presented as follows: * $p < 0.05$

n.s., not significant
Tukey’s test; Australian vs. Hybrid vs. Japanese Wagyu

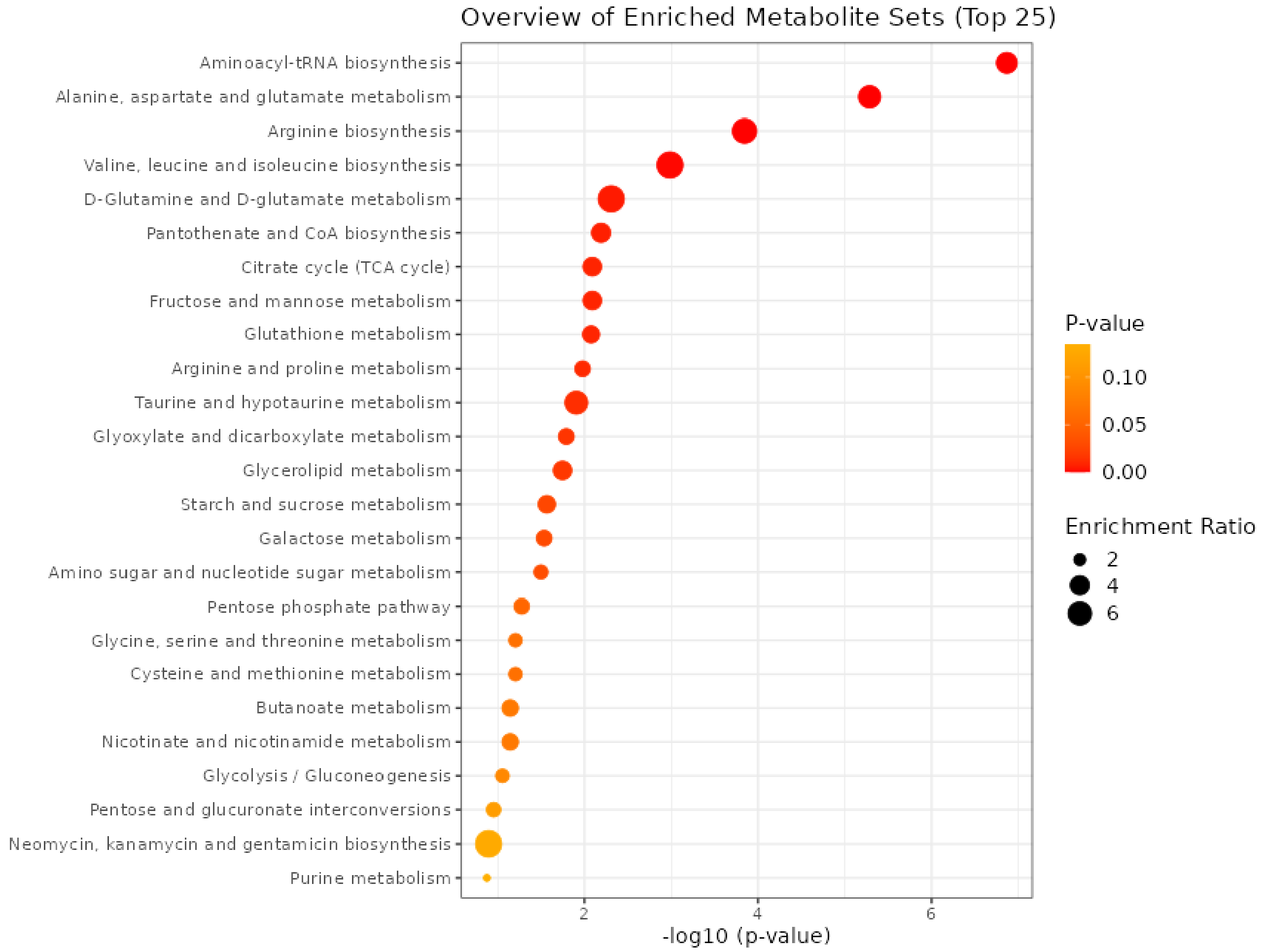

Figure S4. Results of metabolomics data by gas chromatography–mass spectrometry.
Using the Kyoto Encyclopedia of Genes and Genomes pathway database (http://www.genome.jp/kegg/), we identified the metabolic pathways showing marked differences between Japanese and Australian Wagyu cattle.

Plasma conditions	ICP-MS	ICP-OES
RF Power	1.3 kW	1.20 kW
Plasma gas flow	15.5 L/min	15.0 L/min
Auxiliary gas flow	1.55 L/min	1.50 L/min
Neplizer-gas flow	0.95 L/min	-
Neplizer-gas pressure	-	200 kPa
Pump speed	5 rpm	15 rpm

Element	ICP-MS Mass-to-charge ratio (m/z)	ICP-OES Wavelength (nm)
Li	7	
Co	59	
Cu	65	
Rb	85	
Y	89	
Mo	98	
Ag	107	
Cd	111	
Cs	133	
Tl	203	
Internal Standard	115	
Na		589.592
Mg		285.213
P		213.618
K		766.491
Ca		317.933
Mn		257.610
Fe		238.204
Zn		213.857
Sr		407.771
Ba		455.403

Figure S5. Summary of analytical instrument conditions and elemental references for inductively coupled plasma optical emission spectrometry (ICP–MS) and inductively coupled plasma mass spectroscopy (ICP–OES). Elemental concentrations were measured by the standard internal method using ICP–MS and ICP–OES under the measurement conditions presented in the table. As appropriate, mixed standard solutions for calibration curves were prepared by diluting single-element standard solutions (for ICP analysis). Each sample was assayed twice.
